# Capturing structure and function in an embryonic heart with biophotonic tools

**DOI:** 10.3389/fphys.2014.00351

**Published:** 2014-09-23

**Authors:** Ganga H. Karunamuni, Shi Gu, Matthew R. Ford, Lindsy M. Peterson, Pei Ma, Yves T. Wang, Andrew M. Rollins, Michael W. Jenkins, Michiko Watanabe

**Affiliations:** ^1^Department of Pediatrics, Case Western Reserve University School of MedicineCleveland, OH, USA; ^2^Department of Biomedical Engineering, Case Western Reserve University School of EngineeringCleveland, OH, USA

**Keywords:** cardiovascular development, optical coherence tomography, optical pacing, optical mapping, avian models, fetal alcohol syndrome, congenital heart defects

## Abstract

Disturbed cardiac function at an early stage of development has been shown to correlate with cellular/molecular, structural as well as functional cardiac anomalies at later stages culminating in the congenital heart defects (CHDs) that present at birth. While our knowledge of cellular and molecular steps in cardiac development is growing rapidly, our understanding of the role of cardiovascular function in the embryo is still in an early phase. One reason for the scanty information in this area is that the tools to study early cardiac function are limited. Recently developed and adapted biophotonic tools may overcome some of the challenges of studying the tiny fragile beating heart. In this chapter, we describe and discuss our experience in developing and implementing biophotonic tools to study the role of function in heart development with emphasis on optical coherence tomography (OCT). OCT can be used for detailed structural and functional studies of the tubular and looping embryo heart under physiological conditions. The same heart can be rapidly and quantitatively phenotyped at early and again at later stages using OCT. When combined with other tools such as optical mapping (OM) and optical pacing (OP), OCT has the potential to reveal in spatial and temporal detail the biophysical changes that can impact mechanotransduction pathways. This information may provide better explanations for the etiology of the CHDs when interwoven with our understanding of morphogenesis and the molecular pathways that have been described to be involved. Future directions for advances in the creation and use of biophotonic tools are discussed.

## Introduction: a need for tools to assess structure and function of the developing heart

**Figure 1:**
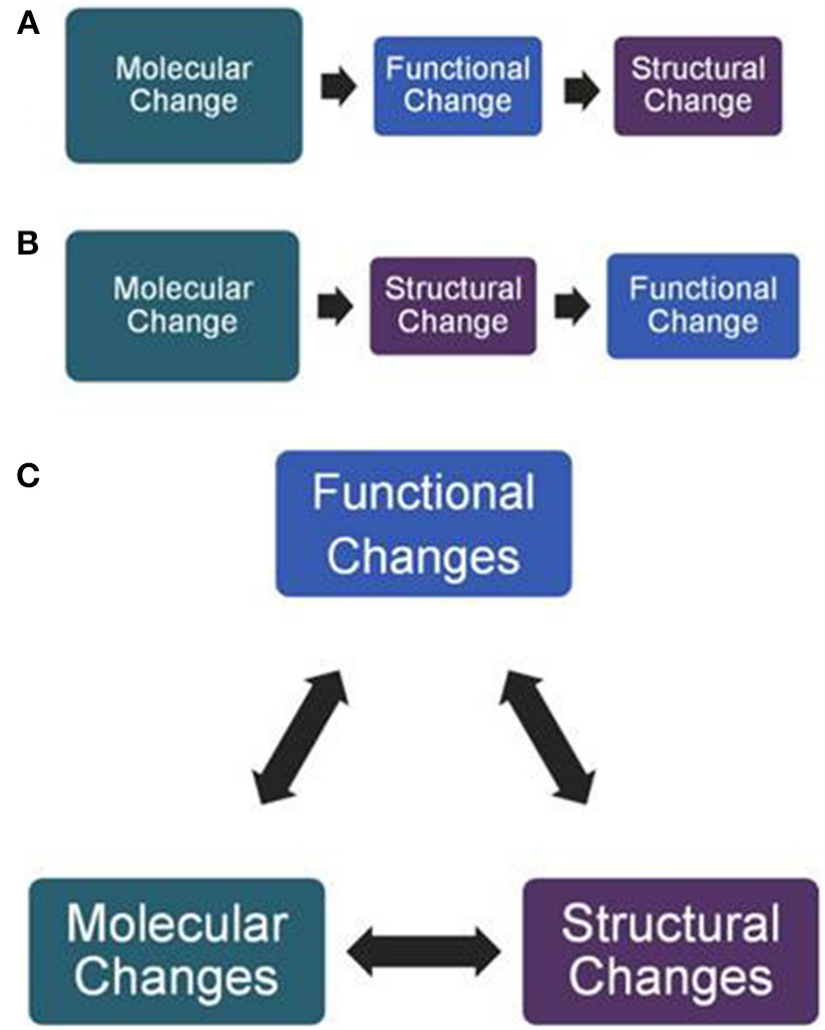
**Interaction of factors that influence heart development. (A)** Molecular changes such as the knockout of ion channels or mutation of sarcomeric proteins are known to alter the function of the heart that then leads to compensatory structural changes in the ventricle. **(B)** There are also examples of molecular changes such as the knockout of extracellular matrix molecules that alter the size and shape of the endocardial cushions that in turn alter shear force. **(C)** However, it is likely more complex in that the factors probably influence each other throughout heart development and an alteration in any one may influence the others. For this reason, it becomes important to obtain and integrate an understanding of all factors. At present, function remains the least understood.

Molecular expression, structure, and function are key factors that likely influence each other throughout development (Figure 1). Our understanding of the molecular/cellular and structural aspects of heart development has far outstripped our understanding of the functional changes. Because the heart begins beating very early, it is capable of imparting biophysical information to embryonic tissues from an early stage. It is therefore essential to capture functional parameters as early as possible and throughout the stages of dynamic morphogenesis. The embryo is highly susceptible and vulnerable to be waylaid from its normal developmental trajectory at these early stages.

The role of biophysical forces in sculpting the developing cardiovascular system is well-accepted. However, accurate measurement of these forces during the genesis of congenital heart defects (CHDs) and sensitively assaying the consequences of these forces is challenging because of the small size, the fragility of the tissues, the continuous contraction, and the complexity of the continuously changing 3-D morphology of the heart. To glean more of this information, we have created and adapted biophotonic tools to probe early as well as late stages of cardiovascular development. These tools allow rapid, thorough, and quantitative analysis of structure and function. The set of tools has undergone continuous evolution as each discovery leads to new questions and challenges to overcome.

We discuss in this review the strengths and limitations of several biophotonic techniques, their current and future potentials, and examples of their use to study an avian model of ethanol-induced CHDs. Emphasis is placed on those methods and applications used by the authors.

## The biophotonic tools

### Optical coherence tomography (OCT)

Optical coherence tomography (OCT) imaging is based upon low-coherence interferometry with high spatial resolution (2–20 μm) and penetration depths (1–3 mm) suited to imaging entire intact embryo hearts at early stages (tubular and looping) without the use of any contrast agents or tissue preparation of any kind (Yelbuz et al., 2002; Jenkins et al., 2007; Garita et al., 2011). Also, OCT requires no contact with the embryo and is non-destructive, allowing for observations of the avian heart of the intact embryo on the yolk cultured in a dish or *in ovo* under physiological conditions. This allows longitudinal studies of the same embryo (Happel et al., 2011; Jenkins et al., 2012).

**Figure 2:**
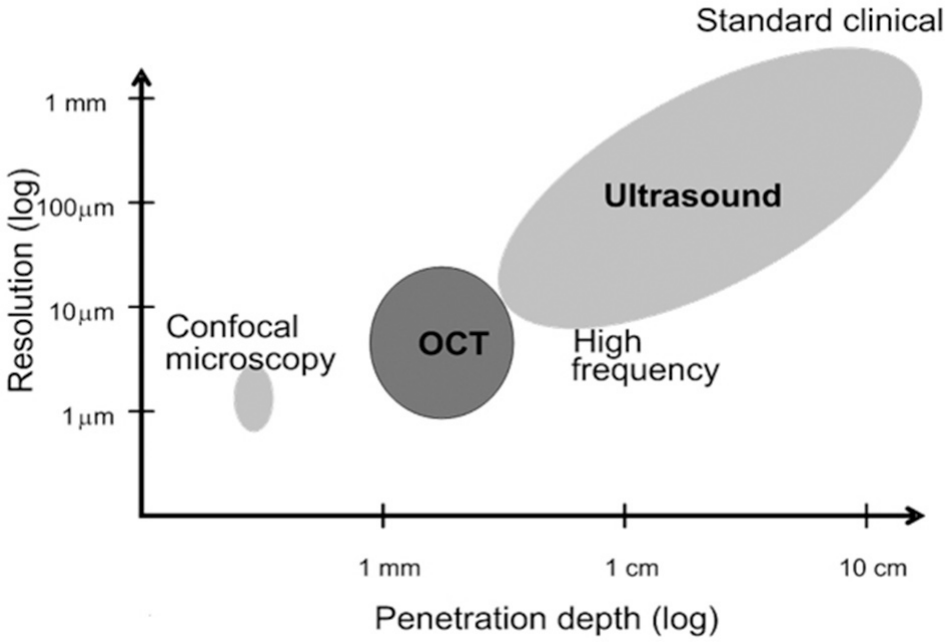
**Confocal vs. OCT vs. Ultrasound**. The resolution and penetration depth of OCT falls between those of confocal microscopy and ultrasound (echocardiography). These parameters make OCT highly suitable for analysis of the embryonic heart (figure modified from Fujimoto, 2002).

OCT is comparable to echocardiography but uses light waves rather than sound waves. In the range of resolution and penetration depths, it lies between echocardiography and confocal microscopy (Fujimoto, 2002) (Figure 2). OCT is used extensively in the ophthalmology setting to non-invasively and rapidly assess eye structures *in situ* (Sohrab and Fawzi, 2013). Intravascular OCT (IVOCT) is also being used in the adult cardiology setting to detect the state of coronary vessel walls via a catheter. With further testing, IVOCT may become useful to assess plaque formation, detect the accumulation of macrophages, identify stent struts, and detect vessel wall dissections (Prati et al., 2011; Tahara et al., 2012; Tearney et al., 2012; Ferrante et al., 2013; Saw et al., 2013). OCT techniques have been and are being developed and adapted by our group and others to study the structure and function of the developing heart (reviewed in Gu et al., 2011; Jenkins et al., 2012).

#### OCT for structural analyses

One of the most basic needs for studying heart development is to capture morphology in a thorough and timely fashion. Many of the classical techniques such as standard histological analysis require time consuming tissue preparation, a high level of skill in serial sectioning, and the ability to analyze three-dimensionally complex structures using two-dimensional images. The preparation and analysis can be expensive and lengthy for large cohorts of embryos.

Because it is much easier to identify and illustrate cardiac defects in 3-D, images of histological sections are often segmented and cardiac structures reconstructed *in silico* (e.g., Newbern et al., 2008; Scherptong et al., 2012; Briggs et al., 2013) (example in movie Figure 1S in Supplementary Material). The challenge of 3-D reconstruction is that sections are likely to be at slightly different angles and rotations or deformed during the collection of sections on slides so that adjustments of the images must be made by eye or by using computer programs to semi-automatically register the images.

Episcopic fluorescent imaging capture (EFIC; Weninger and Mohun, 2002, 2007; Rosenthal et al., 2004; Geyer et al., 2009; Mohun and Weninger, 2012) and related techniques have proven to be extremely useful in reconstructing embryos and analyzing CHDs by avoiding the problems of registration. For EFIC, embryos are embedded in a dark medium to minimize reflected light and sectioned. Each block face is captured digitally as transmitted light or fluorescent images. Interactive 3-D images are reconstructed and analyzed using image processing programs. While superior to standard histological analysis, they are still time consuming even when image collection is automated.

Compared to classical and other methods of histological analysis OCT has clear advantages for the study of early cardiogenesis, particularly for the stages of looping and early chamber differentiation. For avian embryos these would be stages 12–25 determined by the criteria of Hamburger and Hamilton (1992). The comparable stages in mouse embryos would be ED9.5–11.5 (Wessels and Markwald, 2000). One big advantage is that OCT can capture morphology of these early stage hearts with no fixation, contrast agents, embedding, or preparation of any kind. The heart can be scanned immediately after dissection. For certain stages, the avian embryo (up to stage 17–18) heart can be imaged in the intact embryo on the yolk. At later stages (incubation day 3, stage 19–20), when the embryo has turned its head and upper body, the outflow tract (OFT) and part of the right ventricle, can be imaged with OCT. However, another challenge at this stage is that the embryo begins to move intermittently making data collection for 4-D analysis potentially challenging when the goal is high resolution and a large field of view. Later stages can also be analyzed, but the depth of penetration of OCT limits analysis of the intact and unprepared heart to certain vessels and parts of the heart that have been removed from the embryo. Even with these limitations, OCT allows longitudinal analysis of cardiac structure starting from an early stage in development.

Another use of OCT is for staging while the avian embryo is on the yolk. During early stages (day 2 of incubation, Hamburger and Hamilton stages 13–15), counting somites is difficult in these transparent embryos without removing them from the yolk and viewing with transmitted lighting through the embryo. Somites can be scanned without removing the embryo using OCT and the data processed within minutes to obtain the somite number. This number when considered with the other criteria allows for more accurate staging of these rapidly developing embryos without disturbing their development. For later stage embryos, when somite counting becomes difficult because the caudal part of the embryo begins to curve under the body, analysis of OCT scanned fore- and hind-limbs can be used to stage the embryos even if the limbs are at an angle that is difficult to observe in the 2-D plane that is accessible by stereomicroscopy. Staging accurately while the avian embryo is intact in shell-less culture is a great advantage when designing experiments where timing of the application of experimental manipulation has an impact on the outcome. Accurate staging is likely to be most important at early stages of development where critical processes such as heart looping and neural crest development proceed rapidly.

**Figure 3:**
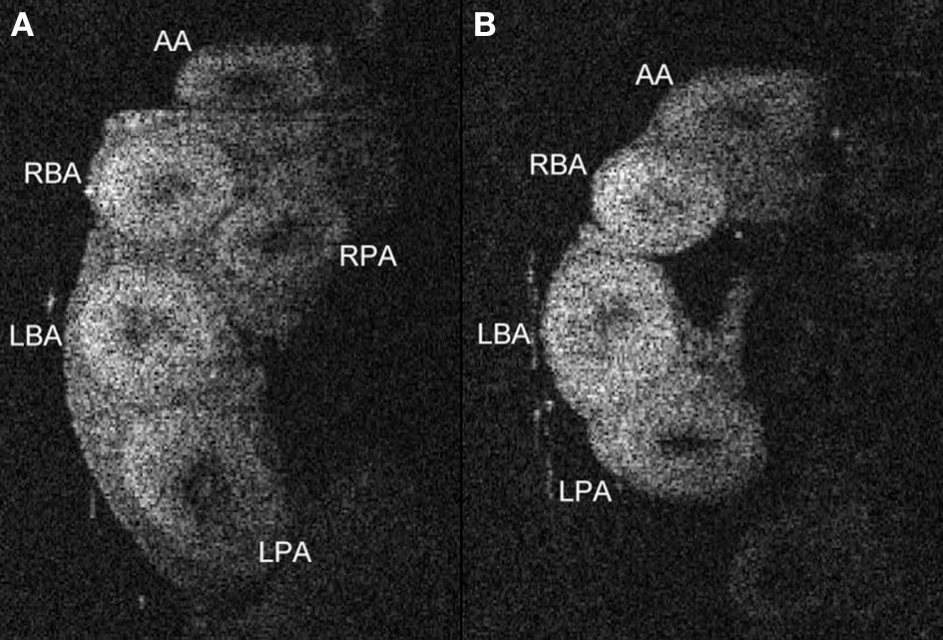
**Anatomy of proximal and distal portions of the outflow tract in in cleared embryo hearts**. OCT images in a plane transverse to the major great arteries of the heart in a stage 34/35 quail embryo allow the identification of the vessels. Comparison of OCT images from untreated **(A)** and ethanol-treated **(B)** embryos illustrates one of the post-septation stage consequences of ethanol treatment at gastrulation, a missing right pulmonary artery (RPA). The dimensions of these vessels such as wall thickness and diameters can be quantified using these OCT images. AA, aortic arch; LBA, left brachiocephalic artery; RBA, right brachiocephalic artery; LPA, left pulmonary artery; RPA, right pulmonary artery.

OCT can also be used to analyze later stage hearts if the tissue is fixed and cleared prior to scanning. One of the simpler clearing processes uses a graded series of formamide concentrations (Kuwajima et al., 2013). After this clearing treatment, older heart tissues are accessible to OCT imaging allowing the analysis of cardiac valve leaflet volumes (Karunamuni et al., 2014), alignment anomalies of the proximal and distal portions of the OFT, and inner and outer diameters of the great vessels (Figure 3). Other more elaborate clearing methods have been introduced for the study of neural tissues and may be useful when adapted to cardiac tissues (Hama et al., 2011; Erturk et al., 2012; Chung et al., 2013; Ke et al., 2013). In combination with structural and functional data collected using OCT at earlier stages of the same embryo, correlation of the severity of early defects and the later CHD outcomes can be made.

**Figure 4:**
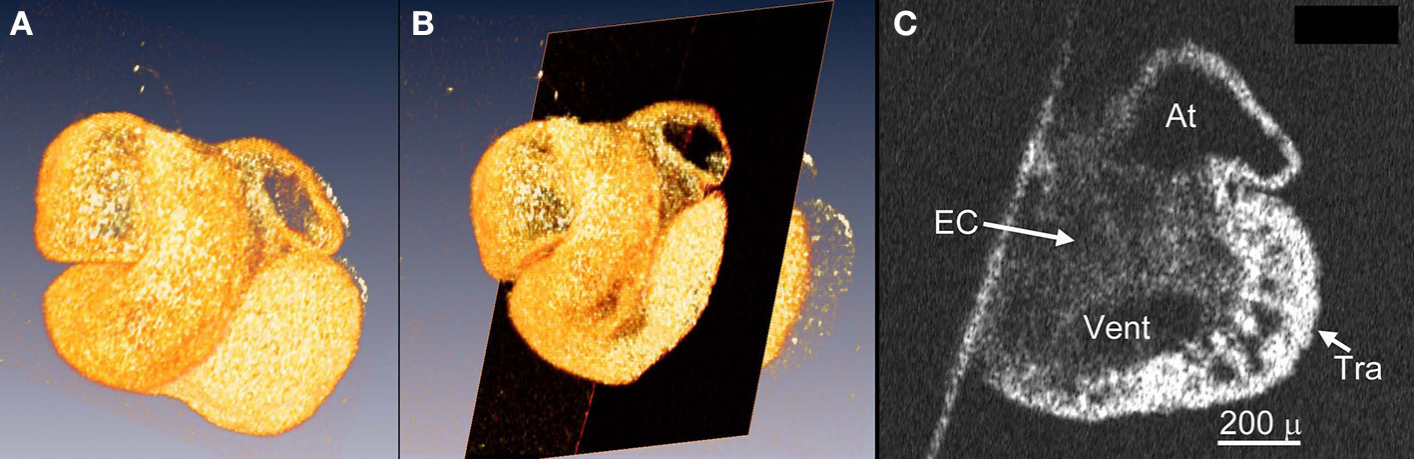
**3-D reconstructions using OCT images can be viewed in any plane of section**. OCT images of a stage E10.5 mouse (C57/Bl6) embryo heart were 3-D reconstructed using the software AMIRA **(A)** and a slice of the heart (black plane in **B**), that is optimal for assessing the endocardial cushions (EC) at the atrioventricular junction and trabeculae (Tra), was chosen to view **(C)**.

OCT images can be used to make 3-D reconstructions of embryonic hearts whether fixed or unfixed (Garita et al., 2011). The value of this basic capability is that the OCT images similar to EFIC images require no registration because the images are acquired from an intact heart and the reconstruction can be rotated and digitally sectioned in any plane for qualitative and quantitative analysis of congenital defects (Figure 4). When hearts are developing abnormally, assessment of particular structures such as the width of the interventricular septum or the volume of the valve may require a different plane of view than for a normal heart. The registered 3-D images easily allow these adjustments.

**Figure 5:**
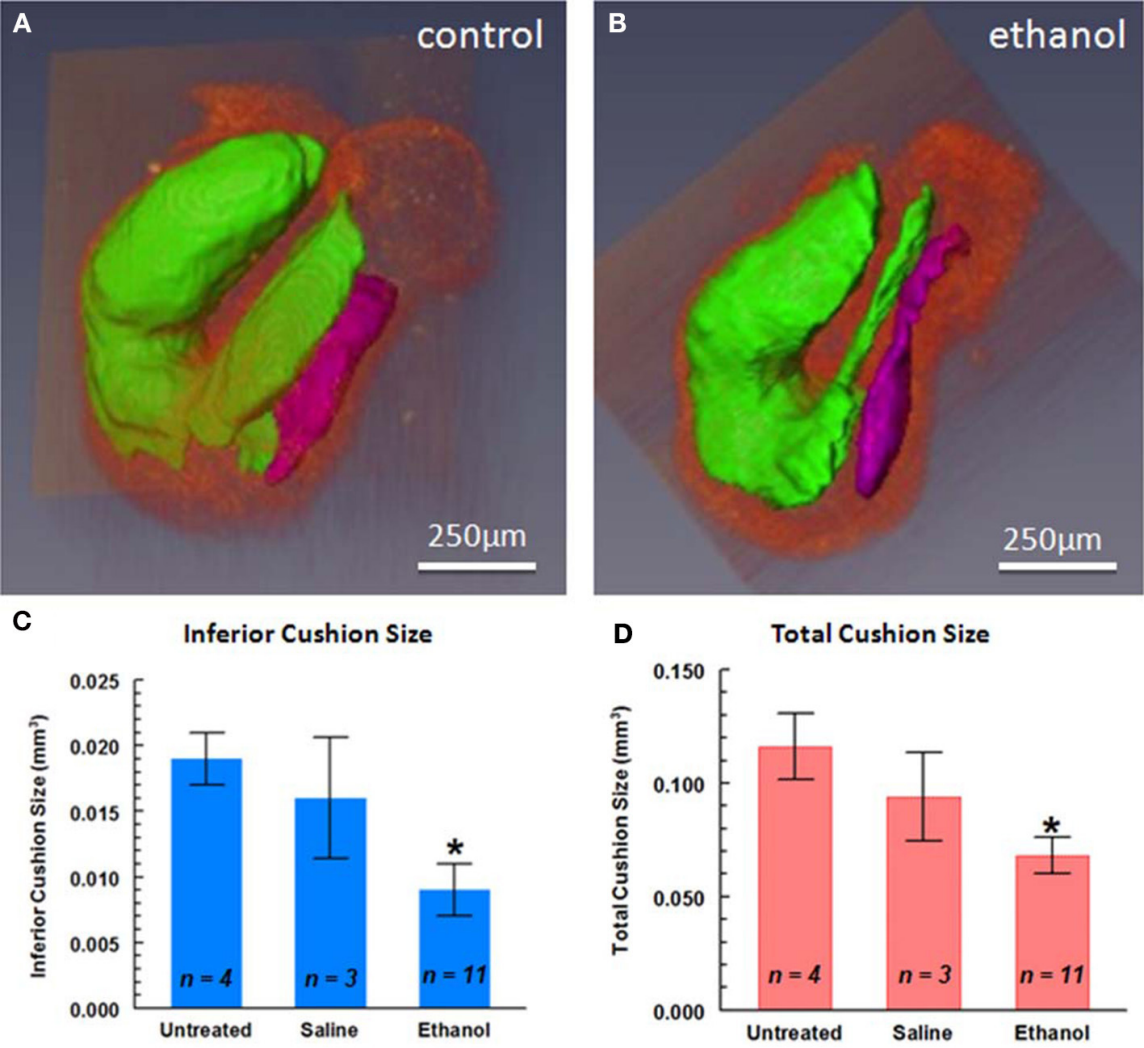
**3-D reconstruction of endocardial cushion volumes of the looping heart**. The major endocardial cushions of **(A)** and ethanol-exposed **(B)** quail embryo hearts were reconstructed from segmented OCT images using AMIRA software. Cushion volumes were smaller in the ethanol-exposed embryos compared to the untreated or saline-treated (vehicle) controls **(C,D)**. ^*^P < 0.05, compared with saline (vehicle) control. Purple, interior atrioventricular cushions; green, superior atrioventricular and cushions in proximal and distal portions of the OFT (from Karunamuni et al., 2014).

OCT images have been particularly useful in the quantification of the size of cardiac structures such as the endocardial cushions (Karunamuni et al., 2014). The 3-D complexity of these structures makes it difficult and time consuming to measure accurately using histological sections. OCT images can be simply followed from adjacent image to image without registration allowing accurate segmentation of cushions and other structures and the calculation of volumes (Figure 5).

**Figure 6:**
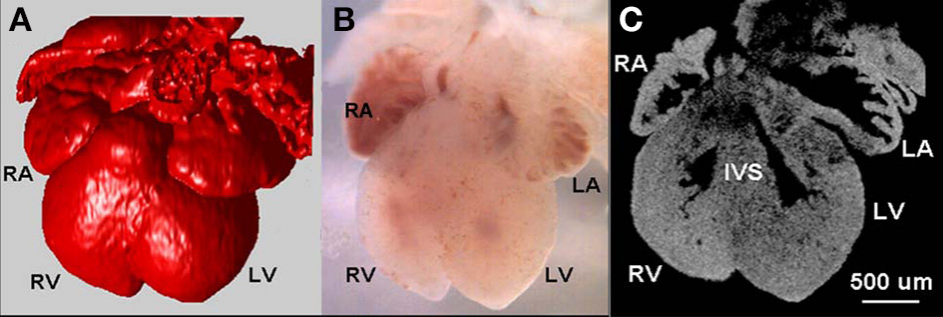
**OCT surface contours of the embryonic mouse heart**. Imaging the E13.5 wild-type embryonic murine heart. 3D reconstruction using OCT images (**A**, red) of the heart taken under a stereoscope microscope with visible light **(B)**. The OCT 2D image **(C)** that can be taken from any plane reveals internal structures. RA, right atrium; RV, right ventricle; LA, left atrium; LV, left ventricle; IVS, interventricular septum (modified from Jenkins et al., 2007).

**Figure 7:**
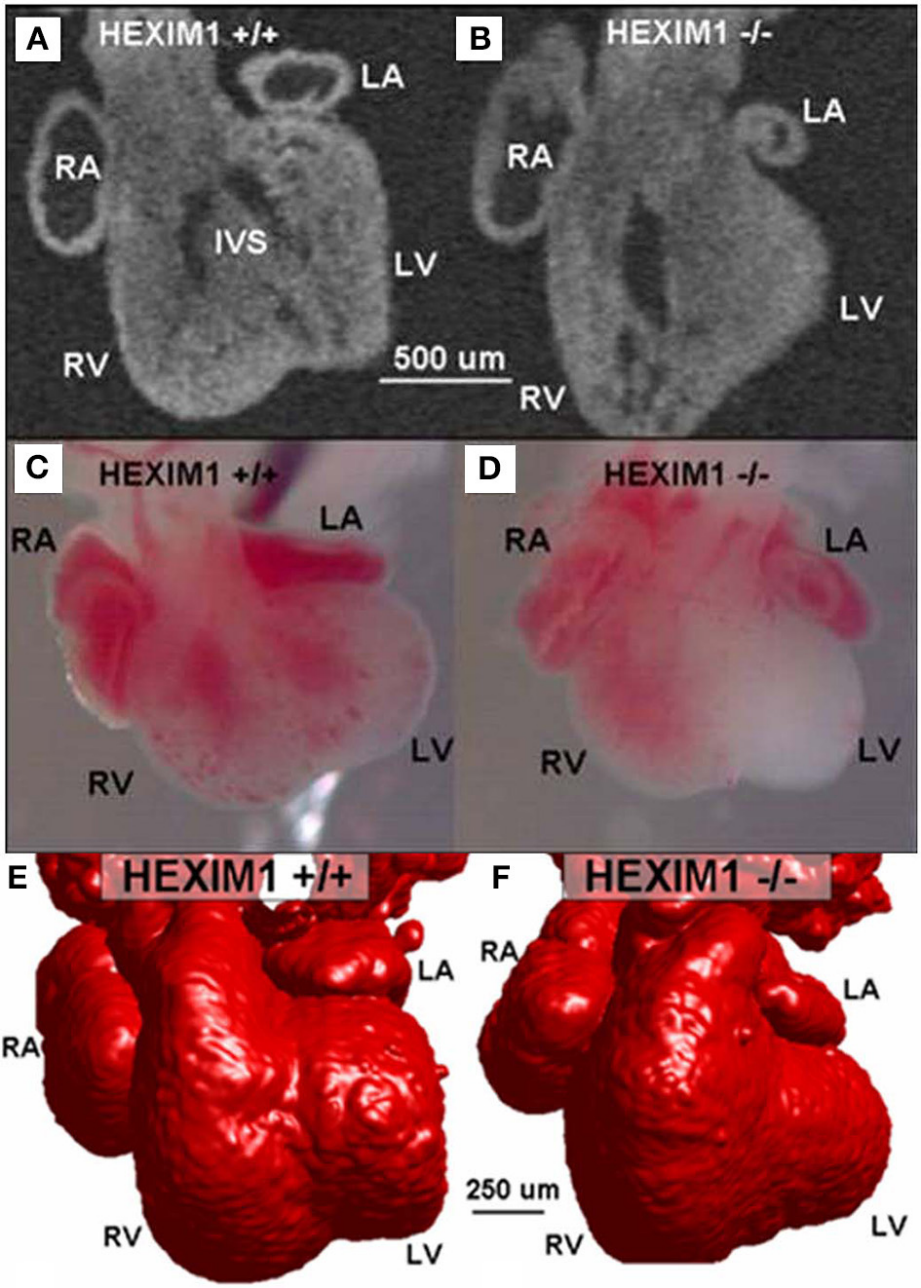
**E13.5 HEXIM1 mutant heart phenotyping (modified from Jenkins et al., 2007)**. 2-D OCT image of a semi-frontal plane of a wild-type heart **(A)** compared to the HEXIM1 homozygous mutant heart **(B)**. Stereoscopic images of wildtype **(C)** and mutant hearts **(D)** indicate little blood in the left ventricle (LV) of the mutant heart. The interventricular septum (IVS) in the wildtype is present **(A)** but in a different orientation than in the mutant heart **(B)**. 3-D surface rendering reconstructions from OCT images (red) of wildtype **(E)** and mutant **(F)** embryo hearts. The left atrium (LA) is smaller in the mutant than in the wild type and the left ventricle (LV) has little of no lumen, while the right ventricle (RV) lumen is enlarged. RA, right atrium; RV, right ventricle; LV, left ventricle; LA, left atrium; and IVS, interventricular septum (from Jenkins et al., 2007).

Reconstruction of OCT images also allows analysis of surface contours of the heart resembling the surfaces viewed by scanning electron microscopy at low resolution. This view emphasizes sulcuses (indentations) between chambers of the heart and can be useful in detecting altered chamber size or the relative alignment of heart structures (Figure 6). OCT images of the HEXIM1 knockout mouse revealed embryo heart ventricular chamber size and alignment abnormalities of proximal and distal portions of the OFT (Jenkins et al., 2007) (Figure 7).

In summary, the capability of using OCT to rapidly collect structural data *in vivo* or in fixed tissues allows the analysis of the consequences of experimental manipulation in large cohorts of embryos in a timely fashion. OCT also allows accurate and quantitative structural phenotyping. The ease of 3-D reconstruction of OCT images and analysis using those interactive reconstructions facilitates accurate phenotyping of external and internal cardiac structures.

### Optical coherence tomography (OCT) for function analyses

#### Doppler OCT (DOCT)

**Figure 8:**
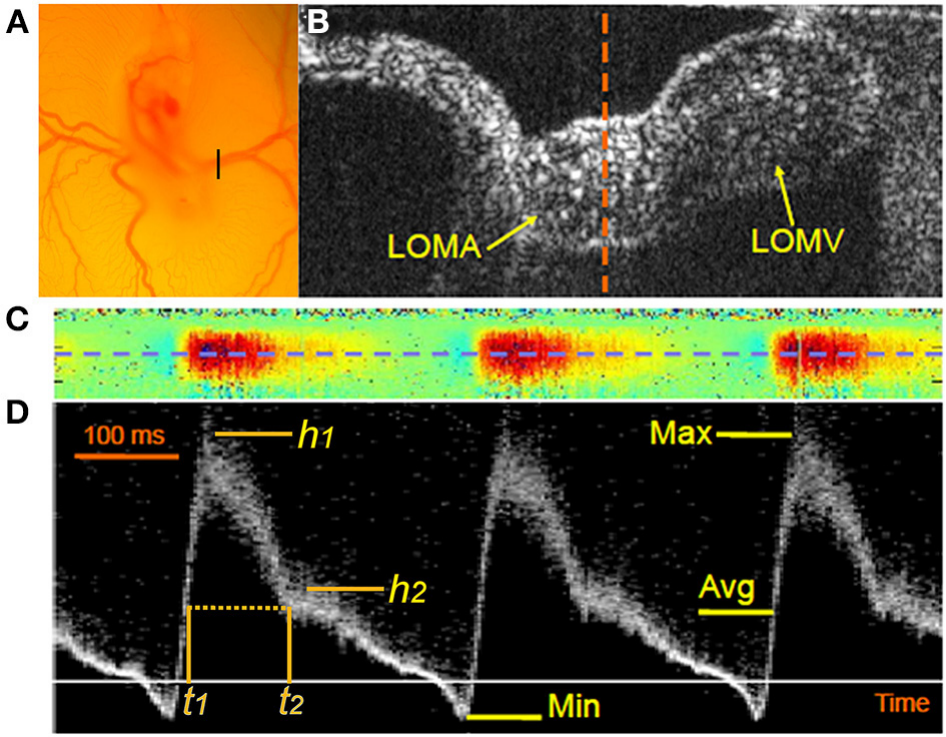
**Dopper OCT of the vitelline artery**. DOCT provides high resolution pulsed Doppler wave forms from the vitelline artery to rapidly capture hemodynamic parameters. **(A)** A stereoscopic view of the stage 17 quail embryo as it site on the yolk reveals the connection of the embryonic circulation with the extraembryonic vasculature in an ex ovo shell-less culture. The black bar marks the site of the vitelline vessels that was scanned for OCT imaging. **(B)** A B-scan OCT image of the vitelline vessel shows a cross section through the left omphalomesenteric artery (LOMA) and the omphalomesenteric vein (LOMV). The dotted line indicates the B-scan plane through the LOMA. **(C)** A colored M-mode Doppler image at the midline of the LOMA (see dashed orange line in **B**). The red color indicates forward blood flow and the blue color indicates retrograde blood flow. **(D)** Pulsed Doppler images at the center of the LOMA (from the blue dashed line in **C**). The magnitude of the signal indicates relative flow velocity with positive values representing forward blood flow and the negative values representing retrograde or regurgitant flow. Max, maximal flow; Min, minimal flow (or maximal retrograde flow); Avg, timed average flow (from Gu et al., 2012).

OCT is a useful tool in identifying blood flow in real time (Figure 8). Doppler OCT (DOCT) works under the similar principle as Doppler ultrasound in that both detect the Doppler frequency shift in signals bouncing back from the blood cells flowing in the heart or blood vessels (reviewed in Leitgeb et al., 2014). OCT however detects the shift in light waves rather than sound waves. Either M-mode (1D scan over time) or B-mode (2D scan over time) Doppler OCT (DOCT) can be used to quickly find and orient the heart region or vessel to prepare to collect data for more detailed live imaging. The images captured are very similar to ultrasound Doppler displays where different colors are used to indicate blood flow away and toward the transducer and a color map with varying brightness/hue/saturation is used to distinguish relative flow rates. The color Doppler can be overlaid on the gray-scale OCT images and displayed in real-time. The additional blood flow information can greatly facilitate identification of target regions where the gray scale contrast may be limited. Unusual flow patterns such as increased retrograde flow, larger or smaller than normal areas of flow, or flow in inappropriate areas can quickly be detected using this method. Doppler OCT can be used for to detect flow parameters (pulsed Doppler), analyze hemodynamic forces, and provide numbers for computer modeling.

#### Pulsed Doppler OCT (DOCT) waveforms

DOCT is only sensitive to movement parallel to the interrogation beam, thus requires angle correction to obtain the absolute velocity information. A simpler application of DOCT is to only collect the relative flow information without any angle corrections, the so-called waveform analyses. Similar to Doppler ultrasound, waveforms can be recorded in either M-mode or B-mode, although the M-mode waveform has a much higher temporal resolution (the line rate of the OCT system can be >100 kHz line rate) and the resulting waveforms are more informative.

Pulsed Doppler ultrasound has been used to assess blood flow waveforms in the embryo (Hornberger and Robertus, 2005; Schellpfeffer et al., 2005, 2007; McQuinn et al., 2007; Oosterbaan et al., 2009). Ultrasound has been used to distinguish flow from the inflow, outflow and primitive right and left ventricles in as early as stage 18 chicken embryos but because of its limited spatial resolution (30 × 75 μm) flow from multiple locations may be found in a particular trace even at this stage. It is certainly not ideal for the study of earlier stage hearts when distinguishing flows from the different regions of the smaller heart is much more problematic. Ultrasound also requires the application of an acoustic medium between the embryo and the transducer such as a gel or liquid medium that could prove invasive for avian embryos and certainly if applied for any length of time (Voronov and Taber, 2002; Voronov et al., 2004; Taber et al., 2010). Doppler OCT is more advantageous for several reasons than ultrasound in analyzing these earlier stages of development due to its higher spatial resolution. No medium is required during scanning. The region of interrogation by DOCT can be small enough to only include the region of interest without contributions from other parts of the tiny heart. When operated in M-mode, DOCT generates pulsed Doppler wave forms at high temporal and spatial resolution to scan the small avian vitelline vessels and allows rapid screening of hemodynamic changes under various perturbation conditions. The analysis of flow patterns in the vitelline vessel by this method can be used to detect alterations of function to identify embryos that that have been affected (e.g., Karunamuni et al., 2014). These identified embryos can then be subjected to the more thorough but more time consuming and more detailed Doppler OCT analysis of flows through the beating heart itself (Gu et al., 2012).

#### Angle-corrected velocity measurement over time

DOCT directly measures the movement in the direction of the beam, and is insensitive to velocity components that are perpendicular to the beam. The measured velocity is therefore always smaller than the true velocity, depending on the angle between the beam and the flow direction (also known as the Doppler angle). If the Doppler angle is known, a correction can be made to obtain the absolute velocity. With most DOCT applications (M-mode or B-mode), the Doppler angle can be obtained by a separate volumetric scan of the same region of interest, and the direction of the blood vessel or heart tube can be established using a 3D rendering of the tissue (Michaely et al., 2007; Makita et al., 2008). With a simple assumption that the blood flow is parallel to the vessel orientation, absolute blood flow velocity can be established after the Doppler angle correction. Unlike the Doppler waveform, which only reports the relative flow over time, the absolute velocity can be used to calculate absolute flow, and is more useful when comparing embryos at different stages or embryos exposed to different conditions (e.g., plus and minus ethanol exposure, hypoxia, or normoxia).

Angle-corrected DOCT can also be extended to 4D data collection, which is the 3D flow profile over time. Since the acquisition is already in 3D, vessel orientation information is already embedded in the dataset and an additional volumetric scan is not required. This method is highly suitable for early heart development when the orientation of the heart tube does not necessarily remain the same during each phase of the cardiac cycle. Normal angle-correction cannot be applied since the angle changes over the heart beat cycle. With image-based registration (retrospective gated imaging), we can reassemble the data obtained over many heart beats into one single beat at very high temporal resolution (>70 volumes per heart heat) (Moss et al., 1957; Gargesha et al., 2009; Bhat et al., 2013). The angle-corrected velocities can then be used to calculate maximal velocity and shear stress on the wall (Peterson et al., 2012).

#### Flow measurement

Doppler angle correction is a necessary step to obtain the absolute velocity with regular DOCT setups. However, to obtain absolute flow, several techniques can be applied to avoid Doppler angle correction. One useful technique is to image the region of interest with volumetric scans, and integrate the Doppler shift in the *en face* plane (Jenkins et al., 2010b; Srinivasan et al., 2010). This approach avoids the Doppler angle correction, but requires 4D data acquisition and either requires cardiac gating or limits the field of view to a smaller region. In addition, the vessels being imaged cannot be perpendicular to the beam. An alternative approach is to use multiple interrogating beams at slightly different angles. To obtain absolute velocity, three independent beams are required, however, for the absolute flow calculation, only two beams are required (Blatter et al., 2013; Peterson et al., 2014). This dual-beam method is more suitable for vessels that are parallel to the surface when the Doppler angle correction is more prone to errors, and the *en face* method is not appropriate. The differences between the Doppler shift in the two imaging beams contains the velocity information perpendicular to the B-scan images (the transverse velocity), and can be used to calculate absolute flows (Peterson et al., 2014).

#### Shear force mapping by OCT by 4D gated shear calculations

One important biophysical force that blood flow induces is shear stress on endothelial and endocardial cells. The candidates for transducers of shear stress include monocilia, cell junctions and associated molecules, the actin cytoskeleton, the glycocalyx, and membrane infrastructure (e.g., lipid rafts) (reviewed in Ando and Yamamoto, 2009, 2013; Drake-Holland and Noble, 2012). Shear stress, however it does so, can provide the signal to endothelial and endocardial and surrounding cells to induce molecular and cellular changes that control critical steps in cardiac morphogenesis such as valve differentiation, septation, and trabeculation (reviewed in Hierck et al., 2007, 2008b; Groenendijk et al., 2007).

Studies using *in vitro* systems have provided evidence that certain levels of shear force influence the presence of endothelial monocilia (Hierck et al., 2008a) and expression of mechanotransduction sensitive genes such as KLF2 (Atkins and Jain, 2007; Novodvorsky and Chico, 2014). Furthermore, studies of embryonic hearts have shown that at certain stages of development or after substantial intervention such as the clipping off of a vessel, changes in the presence of monocilia, and expression of specific molecules can be detected at particular endocardial segments (Lee et al., 2006; Van Der Heiden et al., 2006; Groenendijk et al., 2007; Vermot et al., 2009; Goetz et al., 2014). The challenge is to comprehensively measure shear force *in vivo* and link them to these regionally specific cellular and molecular changes. In the complex *in vivo* environment, it is harder to measure physiological shear force in the highly convoluted endocardial surfaces of the embryonic heart. Even for the “tubular” heart stages when anatomy of the heart is at its simplest, shear stress is likely to vary depending on the region scanned. A number of previous observations clearly indicate that the tubular heart is not a simple endocardial tube within a myocardial tube with cardiac jelly in between (e.g., Nakamura and Manasek, 1978; Bellairs and Osmond, 2005; Manner et al., 2009). The lumen of a quail embryo heart during C-looping (stage 13) is irregular and the cardiac jelly varies in thickness with scalloping in some places due to fibrous attachments of the endocardium to the myocardium (Garita et al., 2011; Liu et al., 2012). Thus, even from the first stages of looping of the tubular heart and probably earlier, the shear stress on the endocardium may vary significantly from region to region.

*In vivo* studies of zebrafish and avian embryo hearts support that there is a complex pattern of shear force and retrograde flow during normal cardiac development and this pattern changes prior to the formation of cardiac defects (e.g., Groenendijk et al., 2004, 2007; Hierck et al., 2007, 2008b; Vermot et al., 2009; Poelma et al., 2010; reviewed in Freund et al., 2012). However, the tools available to measure shear stress are limited. What is needed is technology to measure shear stress and retrograde flow along the length and diameter of the heart tube while it is beating under physiological conditions. 4D DOCT serves that purpose (Peterson et al., 2012).

4D DOCT allows direct measurement of fluid shear stress on the endocardial surface along the length of the endocardial wall of early quail embryo hearts over the heart cycle by using image-based retrospective gating (Liebling et al., 2005; Gargesha et al., 2009) and combining the structural and Doppler data. The shear stress is calculated by taking the velocity gradient normal to the wall of the heart tube and the viscosity of blood. 4D Doppler OCT data sets enable direct measurements of shear stress along the length of the endocardial wall over the entire heartbeat. At each acquired time point in the heartbeat cycle, the velocity gradients normal to the endocardium can be calculated from the OCT Doppler profiles. These velocity gradients multiplied by the dynamic viscosity of the blood result in a 4D shear stress map of the developing heart tube. These shear stress maps allow comparisons of shear values at different regions of the endocardium within the same heart. The shear stress can also be analyzed over time enabling additional metrics such as the oscillatory shear index (OSI). OSI is a metric which quantifies the change in direction and magnitude of the wall shear stress and has been shown to be critical for proper valve formation in zebrafish (Vermot et al., 2009). The resultant shear maps in quail embryos at stage 13 clearly showed high shear stress at the OFT and atrioventricular canal (AVC) endocardium with different levels of shear stress around the diameter of the OFT and AVC, that is on dorsal vs. ventral surfaces and at the outer vs. inner curvatures (Peterson et al., 2012). This study determined that at stage 13 in the quail embryo, the highest shear force (7.7 Pa) on the endocardium was found at the inner curvature side of the outflow and was approximately 4 times that of the outer curvature (2.0 Pa). Movies of the dynamic shear patterns can be created. A limitation of this technique is that for any one session there are areas of the endocardium where valid DOCT data cannot be obtained because the direction of blood flow is perpendicular to the OCT imaging beam. This can be overcome by scanning one specimen at different angles but then the challenge would be to combine the data into one map.

#### OCT and computational modeling

Many functional parameters of the developing heart are still inaccessible or difficult to access by current technology. Nonetheless there is enough information that can be combined to derive the answers. Complex functional parameters impinging on the developing heart can be calculated by combining data from imaging modalities (4D OCT), functional measurements, and computational modeling. Computer simulations can be created using these data and a few assumptions can then help to generate numbers for blood flow and cardiac wall stresses (Rugonyi et al., 2008; Liu et al., 2011). This technique was used to analyze the stage 18 chicken OFT data from several embryos to map region and stage specific stresses and strains.

#### Vascular mapping

Mapping and analyzing blood containing vessel networks that are in one accessible plane, as found on the chorionallontoic membrane on the surface of the yolk of the avian embryo (Vickerman et al., 2009), would not necessarily require OCT mapping. However, most vessel networks are deeper in tissues and are often in more than one plane. Standard methods of vascular mapping require injection of a contrast agent such as a fluorescent marker or ink that has the potential to damage vessels or to change the volume of blood in a small embryo. To be able to map without such interventions would be valuable in longitudinal studies. This is where OCT has proven to be a very useful tool for label-free 3-D vascular mapping in developing embryos.

As mentioned earlier, DOCT can be used to image moving particles and reconstruct the vessel network, however, the Doppler signals are only sensitive to motions parallel to the interrogation beam. To overcome this limitation, new techniques were developed based on the fact that surrounding static tissue generally exhibit static speckle patterns over time, while inside the vessel there are dynamic changes in OCT speckle patterns due to blood cell motion (Mariampillai et al., 2008; Mahmud et al., 2013). This increase in the changes of the speckle pattern can be quantified as variance in the intensity of the pixel over time. The resulting contrast can then be displayed as a vascular map without the use of any contrast agent. This new technique has been used in mapping the vessel network in the retina, in mouse tumor models, and during embryonic development (Jenkins et al., 2012; Kim et al., 2013; Mahmud et al., 2013), with the capability of large fields of view (at least 5 × 5 mm) and high resolution/sensitivity that can resolve capillaries with the right set up. Quail embryos as young as stage 13/14 during early looping heart stages have been mapped. It is sensitive to flows in any direction, with a large dynamic range compared to DOCT. However, it is also sensitive to bulk motions, especially out-of-plane motion. It has been used to track the development of the yolk sac vasculature (Sudheendran et al., 2011).

#### Capturing contraction dynamics by OCT

**Figure 9:**
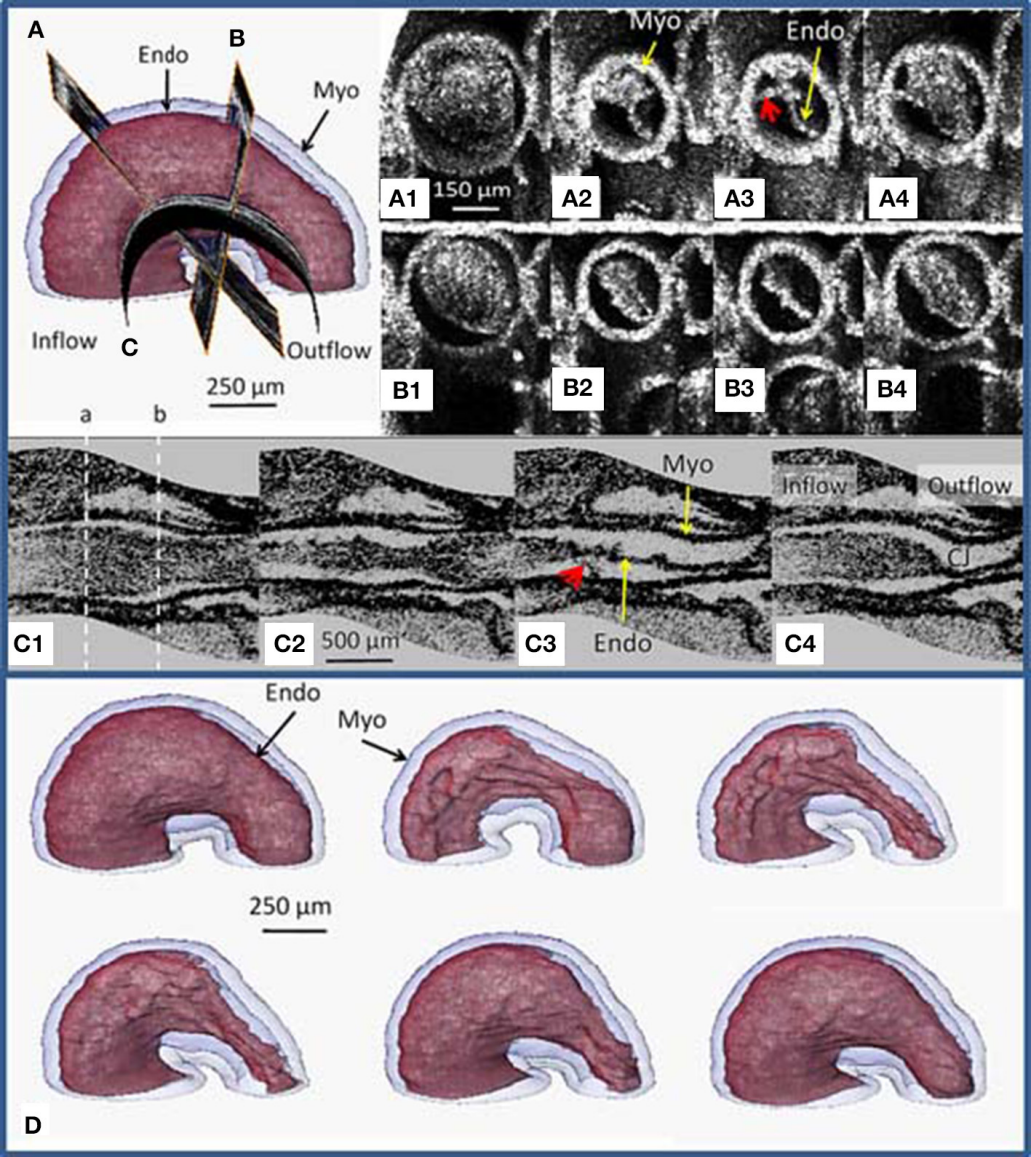
**OCT slices through a stage 13 (early looping) quail heart**. Position of 3 slices **(A–C)** are indicated (upper left). Images were obtained from a time sequence of the beating heart at slice **(A1–A4)** and slice **(B1–B4)** orthogonal to the heart tube. Images from slice a show a complex deformation suggesting tether-like connections between the endocardium and the myocardium (red arrows in **A3,C3**). Images from slice b reveal the eccentric deformation of the tube. Images from the curved longitudinal slice **(C1–C4)** through the center of the heart tube reveal the non-uniform morphology of the heart tube tissue layers including the scalloped endocardial cushions. In **(C1)** the dotted white line indicates where slice **(A,B)** were taken. Surface renderings **(D)** of segmented endocardium (red) and myocardium (blue) at different times during the cardiac cycle reveal the endocardium (Endo) folds into longitudinal ridges as the myocardium (Myo) contracts. Endo, endocardium; Myo, myocardium (from Jenkins et al., 2012).

With image-based retrospective gating, OCT is able to generate 4D data sets of beating hearts at >70 volumes per heartbeat. This is fast enough to investigate dynamic parameters related to normal and abnormal heartbeats. With OCT, each layer of the heart structure can be segmented (myocardium, endocardium, cardiac jelly, or cardiac cushions), and the heart can be viewed in any orientation and plane (Manner et al., 2008, 2009). Particularly useful is the ability to view even curved slices down the middle of the heart tube (Figure 9, Jenkins et al., 2012). In addition to the parameters commonly derived with other techniques (e.g., ejection fraction), contraction dynamics such as contractile wave propagation or cardiac jelly redistribution can also be investigated (Garita et al., 2011). We showed that the contraction pattern and cardiac jelly movements of a stage 13 avian embryo predicted the future location of the AV and OFT cardiac cushions. Detailed flow and wall stress and strain can also be analyzed using OCT imaging and computational modeling (Liu et al., 2011; Li et al., 2012).

#### Tissue property measurements of stiffness and elasticity

OCT based elastography (OCTe or OCE) is where a separate mechanical perturbation is applied to the tissue, and the response of the tissue is measured with OCT. This allows visualization of the mechanical properties of the tissue with a resolution similar to that of the OCT instrument (Schmitt, 1998). Applications include any tissue where the mechanical properties of the tissue are not well-understood and are not amenable to traditional destructive mechanical measurement methods such as stretch or indentation testing. This is especially true in tissues that are complex in nature, because different layers or regions in the tissue need to be assessed independently. This is also true for embryonic tissues that can be easily damaged during mechanical manipulation. In the former situation elastographic imaging has already shown promise in providing quantitative results that help define the nature of tissues in a complex scenario.

OCE has been used extensively to study the properties of the adult cornea and arteries (Rogowska et al., 2006; Ford et al., 2011; Kennedy et al., 2011; Nguyen et al., 2014a,b) to improve surgical outcomes and aid in the basic understanding of these very complex tissues. Elastographic imaging has not been used in embryonic tissues but theoretically could provide much needed information about the properties of delicate developing valve and vessels that are not readily accessible by other techniques.

### Optical coherence microscopy (OCM)

**Figure 10:**
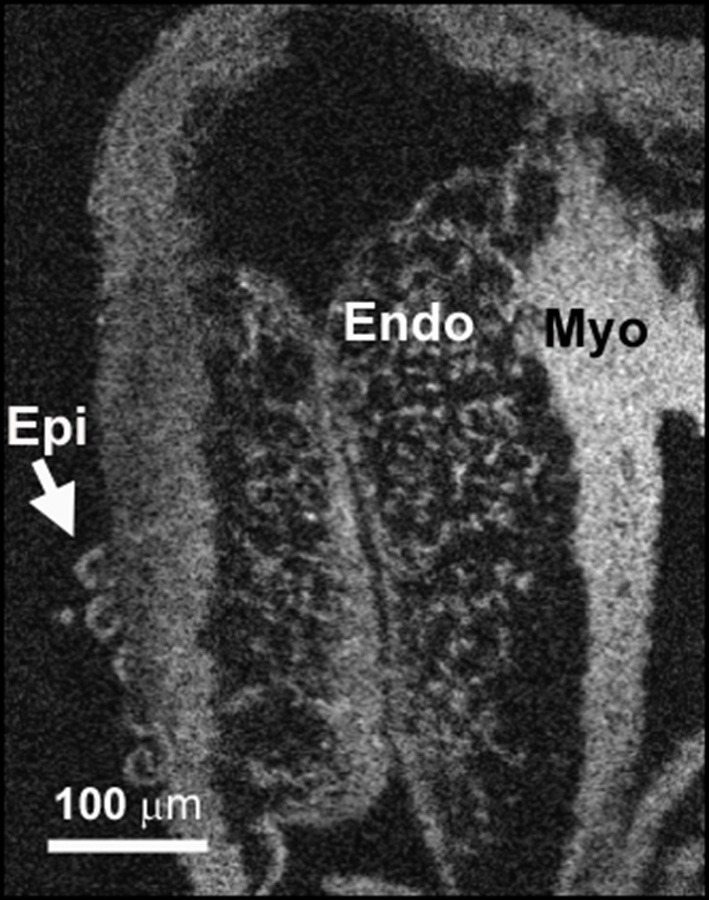
**OCM allows visualization of cardiac tissue morphology**. OCM images have the resolution to reveal important details of cardiac tissue morphology. In this image of the atrioventricular junction of a fixed stage 18/19 quail embryo heart, the endocardium can be easily distinguished from the myocardium (Myo) and epicardial cells (Epi) that have just begun to grow over the myocardium. Individual mesenchymal cells can be detected within the endocardium (Endo) and the degree and location of the mesenchymal content within the endocardium can be assessed.

Optical coherence microscopy (OCM) is based on similar principles as OCT but with the use of higher numerical aperture (N.A.) objective lenses, enabling higher lateral resolution (<2 μm lateral) (Aguirre et al., 2010; Zhou et al., 2010; Lee et al., 2013). The tradeoff is that OCM generally has a smaller field of view (<500 × 500 μm) and less penetration depth (300–500 μm) than OCT. OCM is better suited for imaging small regions of the embryonic heart when higher spatial resolution is desired. The higher resolution of OCM allows visualization of details such as the fibrillar substructures within the extracellular matrix of the cardiac jelly (Garita et al., 2011) or the individual mesenchymal cells within cardiac cushions (Figure 10). This is a level of detail that approaches light microscopy without the need for embedding and physically sectioning the tissue. This reduces processing time, accelerates data acquisition, and minimizes distortion when 3D parameters are measured. The current limits of OCM scanning depth confines the usefulness of OCM to the analysis of young embryo hearts or to regions close to the surface of the heart. An alternative is to prepare and analyze tissue slices that are no more than 0.5 mm in thickness and reconstructing the data sets.

In addition to providing single *en face* images at high resolution similar to regular microscopy techniques, OCM is also capable of providing high resolution 3D volumetric data. Given the short focal length of OCM, the focal range of regular OCM is limited, generally around 20–30 μm around the depth of focus. To obtain volumetric data of sufficient axial distance, the sample has to be physically translated and the resulting multiple datasets registered and merged into a single dataset. An alternative solution is to use non-Gaussian beams to achieve extended focus into the tissue (Leitgeb et al., 2006). This latter method has been used to analyze blood flow in adult brain tissue (Bouwens et al., 2014).

### Optical mapping (OM)

Although not directly considered mechanotransduction, the activation of electrical impulses within the heart initiates contraction of cardiomyocytes that controls the propulsion of blood and potentially the movement of other extracellular fluids. Thus, assessment of cardiac impulse initiation and conduction is important in analyzing the coordination of the complex contraction patterns throughout the developing heart that have a profound effect on mechanical properties throughout the heart and vasculature such as stretch, strain, pressure, and shear force. Another consideration is that calcium fluxes associated with electrical activation is critical for signaling early steps in cardiogenesis (Linask and Linask, 2010). Electrical impulses are also implicated in regulating cell shape and consequently cardiogenesis (Chi et al., 2010). An interesting aspect of cardiac function that can be analyzed by capturing electrical activation data simultaneously with contraction of the myocardium is excitation-contraction (E-C) coupling. This interval between electrical stimulation/activation and muscle contraction could have a significant impact on the function of the heart.

Detecting electrophysiological parameters of the tiny developing heart is possible using pulled glass electrodes and optical mapping (OM) using voltage sensitive dyes. The pattern of electrical impulse conduction has been tracked in the past using two or more electrodes (De Jong et al., 1992; Chuck et al., 1997) and remains the gold standard for action potential morphology. However, OM has clear advantages in that action potentials can be captured from many regions of the heart simultaneously revealing the complex patterns over the heart, making it the recording method of choice for most cases for analyzing activation patterns or conduction velocity. OM has been used for many years to analyze activation patterns in the intact adult heart (reviewed in Efimov et al., 2004; Lee et al., 2012). OM generally uses voltage sensitive dyes that are lipophilic and integrate into membranes and exhibit changes in fluorescence properties that are linearly related to the changes in membrane voltage. The most commonly used voltage-sensitive dye in cardiac OM is the styryl dye, di-4-ANEPPS (Fluhler et al., 1985; Loew et al., 1992), which exhibits a shift in spectrum based on a molecular Stark effect (Loew, 2011). By the simultaneous detection of these voltage changes, action potential origin, velocity and direction can be analyzed. OM is ideal for analysis of the electrophysiological properties of the embryonic heart because it allows for non-contact ultra-high resolution detection of action potentials from the heart surface.

For OM of the intact larger adult hearts, the heart is isolated and perfused with a membrane bound voltage sensitive dye in a Landendorff preparation. The motion of the heart is suppressed with the application of drugs to inhibit contractions. The most commonly used drugs act either by stabilizing the myosin-ADP-Pi complex (2,3-butanedione monoxime blebbistatin) (Herrmann et al., 1992; Kovacs et al., 2004) or altering the kinetics of actin polymerization [cytochalasin D (Kovacs et al., 2004; Shoji et al., 2012)]. The heart surface to be analyzed is sometimes flattened against a pane of glass to reduce the amount of tissue that is out of focus and the data captured using an EMCCD camera.

For OM analysis of embryonic hearts, the heart is isolated and incubated in a solution of the voltage-sensitive dyes and the motion is suppressed as described above for adult hearts. OM enables the detection of sites of initial activation of the avian tubular heart (Kamino et al., 1981; Sakai et al., 1983) and the creation of isochrone maps to follow patterns of conduction in avian and mammalian embryo hearts (Chuck et al., 2004; Rothenberg et al., 2005a,b; Bressan et al., 2013). It has been used to study the early stages of heart development in normal and mutant zebrafish (Chi et al., 2008, 2010; Panakova et al., 2010; Sabeh et al., 2012). OM recordings of the heart were initially taken using small arrays of photodiodes (Hirota et al., 1979; Fujii et al., 1980, 1981a,b,c; Kamino et al., 1981). Technical advances have enabled high-resolution spatial and temporal recordings over large fields of view using either larger photodiode arrays with many elements or high-sensitivity CCD cameras (reviewed in Herron et al., 2012).

One key challenge using OM on embryonic hearts is that the signal-to-noise ratio from the embryonic hearts is significantly lower than in adult heart, due to the small size and thinner tissues. Sensitive cameras, super-bright LED light sources, and signal processing algorithms have made OM possible even at the earliest stages of cardiac development, when the first cardiomyocytes begin to activate electrical impulses.

**Figure 11:**
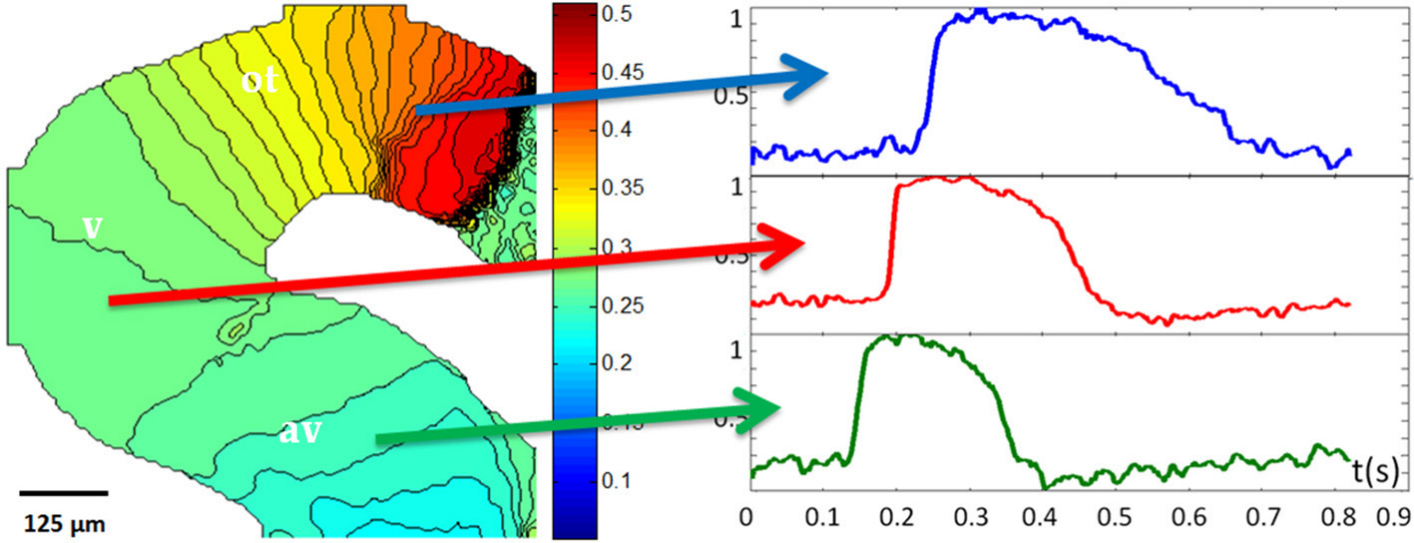
**Optical mapping**. An isochrone map (color map in units of seconds) of a stage 14/15 quail embryo heart indicates that the myocardium of the atrioventricular canal (AVC) has slower conduction properties than the ventricle (V). The outflow tract (OFT) has the slowest conduction of all three regions that were mapped. Action potential profiles from the AVC (green arrow and trace), V (red arrow and trace), and AVC (blue arrow and trace) after temporal averaging of the trace were plotted against a normalized y scale. These traces show differences in action potential morphology depending on the region (adapted from Ma et al., 2014).

Our group has obtained and analyzed OM data from early avian embryo hearts at looping stages (e.g., stage 14) to create high resolution activation (Figure 11) maps. In addition with analysis of the action potentials it is possible to obtain conduction velocity and action potential duration (APD) maps to expand our understanding of the electrophysiology of the developing heart. Even in the tiny embryonic tubular heart, the weak electrical signals can be processed and analyzed to comprehensively follow these electrophysiological properties.

Previous studies using OM have addressed the transition in conduction patterns in developing chicken and mouse embryo hearts by detecting activation. During the early stages of development from the tubular to late looping stages, chicken embryos have a homogeneous and slow conduction pattern with contraction resembling peristalsis (Patten, 1949; Kamino et al., 1981; Hirota et al., 1985a,b, 1987; Bressan et al., 2013). The conduction starts from the inflow in a region that can be considered the primitive sinus node area and moves to the outflow. As chambers differentiate, the heart attains an alternating pattern of slow and fast conduction but the conduction still moving from the top of the ventricle or base to the tip of the ventricle or apex (Patten, 1949; Paff and Boucek, 1962; Irisawa et al., 1965; De Jong et al., 1992; Chen et al., 2010). At the time of ventricular septation when the heart reaches its four-chambered structure, the conduction pattern of the ventricles gradually transitions to the mature apex-to-base pattern presumably because of the preferential conduction through the maturing ventricular conduction system (Chuck and Watanabe, 1997; Chuck et al., 1997, 2004; Watanabe et al., 2002, 2003; Rothenberg et al., 2004, 2005b). The timing of this transition can be altered by experimental interventions that change hemodynamic parameters (Reckova et al., 2003; Sankova et al., 2010). In the initial studies of embryonic mouse and rabbit heart conduction patterns, OM images indicated that the apex-to-base conduction pattern appeared much earlier before the end of ventricular septation (Rentschler et al., 2001; Myers and Fishman, 2003; Rothenberg et al., 2005a). Subsequent OM studies have either not directly addressed nor resolved the issue of the difference in timing of the emergence of the apex to base conduction pattern between mouse and avian embryo hearts (Sedmera et al., 2004; Chen et al., 2007). A potential explanation has been recently proposed regarding the “primary ring” that separates the primitive right and left ventricles and may be responsible for activating the ventricle even prior to the maturation of the ventricular conduction system (Sankova et al., 2010, 2012). Another group has proposed a related atrioventricular ring (AVR) as being similarly involved (Valderrabano et al., 2006; Chen et al., 2007). Further studies on comparing the early stage mouse and avian embryo hearts would be of value especially if a more comprehensive analyses of various electrophysiological parameters is used in addition to activation mapping.

As informative as OM is, it is not currently suited to longitudinal studies of the same subject. Introduction of a voltage-sensitive dye to the heart *in vivo* is difficult, and in the embryonic heart, it is likely to affect development. There are also some genetically expressed optical reporters of membrane voltage that are in development for neural applications (Jin et al., 2012; Kralj et al., 2012), though none currently responds with sufficient speed or signal-to-noise ratio for use in the embryonic heart. Dealing with cardiac motion is also problematic *in vivo* as abolishing contraction by the E-C uncoupler is likely to alter development. While the E-C uncoupler can be washed out, removing it completely so as to restore normal contraction strength is difficult if not impossible. Algorithms for removing motion artifacts by image registration have been used in the analysis of adult hearts with some success (Rohde et al., 2005), but have not been validated in embryonic hearts which can have more complex movements.

### OM integrated with OCT

As mentioned above, two-dimensional (2-D) OM can provide parameters such as action potential morphology (activation time, upstroke velocity, APD), activation sequence and conduction velocity. While action potential morphology and general qualitative activation sequence measurements are not affected by a 2-D projection on the 3-D structural map, the accuracy of quantitative conduction velocity can be severely affected especially when mapping the earlier developmental stages. During these looping stages the embryonic heart is not flat but a complex three-dimensional tubular structure that is undergoing rapid contortions during its morphogenesis. If the embryonic heart is flattened against glass, as is done when OM adult hearts, the embryonic heart may be damaged and the contact with the glass itself can result in introduction of artifacts as a result of mechanical stimulation and hypoxia where the glass limits gas exchange. A potential solution to overcome the difficulty of using OM during stages of rapid morphogenesis is to simultaneously collect OCT and OM data and integrate the 2-D OM data by overlaying it on a 3-D structural map.

Our team has developed an integrated OCT/OM system for imaging both the structure and electrical activity of the embryonic heart (Ma et al., 2014). The OM portion of the integrated system adopted a high intensity broadband LED light source and high numerical aperture objective lenses so that more light can be delivered to the sample. The OM system is capable of recording 128 × 128 pixels at a maximum frame rate of 500 Hz. The OCT system has axial and lateral resolution both approximately 10 μm in air. The OCT operating speed is 47 kHz line rate. The OM portion of the system images the sample from the top while the OCT portion of the integrated system images from the bottom through a transparent coverslip, which is the bottom of the imaging chamber. OCT is capable of imaging through the early embryonic heart to acquire its full 3-D structure.

**Figure 12:**
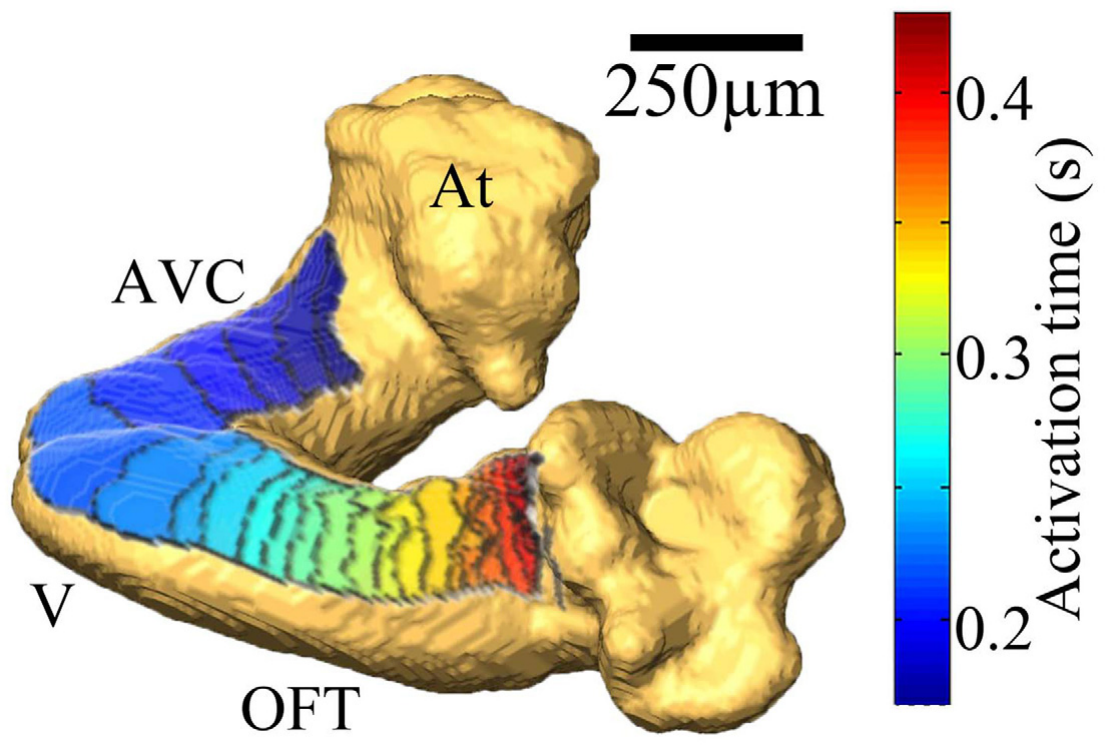
**Integration of OM and OCT data**. 2-D electrical activation map was overlaid on the 3-D OCT surface rendering of a stage 13/14 quail embryo heart to correct for the complex surface structure of the looping heart. The color map blue to red represents the sequence of activation from early to late. Each isochrone represents 10 ms (from Ma et al., 2014).

3-D OCT volume images are registered to the OM image and then segmented to derive a height map. This height map provides 3-D coordinates for each pixel in OM images. We have developed a 3-D conduction velocity correction algorithm based on the 2-D activation time gradients and the 3-D coordinates of each pixel. Most pixels required a correction between 0 and 20%. Depending on the orientation of the embryo heart, 12–41% of the pixels required corrections of >20%. The end product is a 2-D activation map overlaid on the 3-D OCT surface rendering and 3-D-corrected conduction velocity maps (Figure 12).

The OCT/OM integrated system and 3-D conduction velocity correction algorithm improves the accuracy of quantitative conduction velocity measurements and reduces experimental variability due to the irregular heart morphology and orientation of the scan for both normal and abnormal hearts. Simultaneous acquisition will allow directly co-registered structural and electrical information that will make possible the study of how important features of the embryonic heart and conduction system influence each other.

### Optical pacing (OP)

Optical pacing (OP) is contact-free technology that enables 1:1 pacing of the embryonic and adult heart with pulsed infrared light without any genetic manipulations (Jenkins et al., 2010a, 2013). This technology was adapted from a method to activate neural tissues (Wells et al., 2005a,b). The specific mechanisms for optical stimulation are still being explored, but clearly the induced thermal gradient is involved where both membrane capacitance and mitochondrial calcium transients have been implicated (Dittami et al., 2011; Shapiro et al., 2012; Liu et al., 2014a). In contrast, optogenetics requires genetic engineering of cells to express microbial opsins to sensitize the cells to light (reviewed in Fenno et al., 2011; Yizhar et al., 2011). While well-tolerated by many cell types, optogenetics requires the administration of constructs to the cells that may in itself disturb development. OP provides a viable alternative to traditional electrical point stimulation that has significant limitations when used in embryonic hearts, including being likely to cause tissue damage due to contact and high charge densities around the electrode tip, generating a relatively large electrical artifact that interferes with electrical recordings, and having a limited spatial precision. In contrast, OP requires no contact with the heart, creates no electrical artifact, and allows pacing of embryonic and adult hearts without tissue damage (Jenkins et al., 2010a, 2013; Wang et al., 2014). It can be used to pace the heart in intact embryos as well as in dissected hearts. Avian embryo hearts with a heart rate of 0.5–1.5 Hz can be optically paced at 3 Hz or higher for at least many minutes at a stretch. The limits of the OP parameters that can be used on embryo hearts are currently under investigation. For mouse embryo hearts that are particularly difficult to study after dissection, OP may be useful to normalize the heart rate during OCT or OM analysis. Standardizing the heart rate during OM is important because of the frequency-dependent nature of many electrophysiological parameters. The pacing beam can be focused to such a small area [12 μm spot (Wang et al., 2014)] that single cell pacing may be possible. This capability would be useful in pacing cells and tissues in culture to study impulse conduction and activation mechanisms. The potential exists as demonstrated in neural systems (Duke et al., 2013) to also use pulsed infrared light to inhibit activation.

The analysis of mechanotransduction mechanisms requires experimental manipulation of functional parameters. A number of studies have already shown that major disruption of cardiovascular function by constricting the aorta or other large vessel, tying off part of an atrial chamber, clamping vitelline vessels, or occluding the inflow or outflow results in obvious CHDs (e.g., Hogers et al., 1997, 1999; Tobita and Keller, 2000; Sedmera et al., 2002; Hove et al., 2003; Lucitti et al., 2006). Likewise inactivation of the heartbeat by genetic manipulation also results in CHDs and early *in utero* death (Bi et al., 1999; Koushik et al., 2001; Fritz-Six et al., 2003). While informative, these types of disruptions do not reflect the more gradual and subtle changes that are likely to occur during the development of most CHDs. More subtle interventions have been attempted by increasing the viscosity of the blood by injection of hydroxyethyl starch (hepastarch) (Lucitti et al., 2007) and genetic manipulation of zebrafish to decrease hematopoiesis and thus reduce the concentration of red blood cells which in turn reduces shear force (Vermot et al., 2009).

OP provides a new way to precisely perturb embryonic cardiovascular function by altering heart rate *in vivo*. This may allow us to mimic more closely the subtle changes in functional parameters that precede and contribute to the development of CHDs.

## Application of biophotonic tools to study the genesis of CHDs in a model of fetal alcohol syndrome (FAS)

### FASD and FAS

Congenital defects due to prenatal ethanol exposure constitute a major public health issue. Fetal alcohol spectrum disorder (FASD), the umbrella term for deficits caused by pre-natal ethanol exposure, is considered a worldwide epidemic with an incidence of 1% of live births (May and Gossage, 2001; CDC, 2009). Estimations of the prevalence of FASD in school age children in the USA and Western Europe are as high as 2–5% (May et al., 2009). These higher estimations however do not account for miscarriages and stillbirths that may have resulted from ethanol exposure, thus likely underestimating the negative prenatal effects of ethanol exposure (Bailey and Sokol, 2011).

When considering FAS, a defined subgroup of FASD encompassing the most severe of the spectrum of defects, there are 2–7 cases diagnosed per 1000 live births in the USA. Among FAS individuals, the incidence of CHDs, such as ASDs, VSDs, pulmonary stenosis, valvular defects, and defects of proximal and distal portions of the OFT (so called “conotruncal” defects), was at least 20% (Burd et al., 2007). The Burd et al. (2007) literature review of 29 papers reported 28.6% as the rate of comorbid CHD and FASD. These incidences of comorbidity are likely to be underestimations due to ascertainment bias and underdiagnosis as well as misdiagnosis of FAS/FASD as autism. There would likely be selection bias because the most severe forms of FAS that result in an aborted embryo or fetus are most likely to have CHDs and would not be counted.

While neurodevelopmental deficits are the main focus of concern for individuals with FAS/FASD, there are at least two important reasons for investigating CHDs that result from ethanol exposure. (1) The types of CHDs resulting from FAS can be severe and often require surgery and a lifetime of medical care and (2) abnormal cardiac function can have a global impact on the embryo, affecting neurodevelopment, placental growth and development, and growth of vessels that are critical for normal development of other embryonic tissues.

Animal models of FAS/FASD have been useful in probing the mechanisms for ethanol-induced congenital defects and in investigating potential preventative therapeutics such as folic acid, choline, and myoinositol (Smith, 1997; Sant'Anna and Tosello, 2006; Serrano et al., 2010; Wilson and Cudd, 2011; Ballard et al., 2012; Cole et al., 2012).

We are studying a quail model of FAS in which a large proportion of the embryos that survive to septation stages (>50%) exhibit CHDs. A single ethanol exposure of the quail embryo at gastrulation [stage 4; (Hamburger and Hamilton, 1992)] models a single bout of binge drinking (4 or more standard drinks on one occasion) at approximately 2 weeks of human gestation. Using this avian model we are testing the hypotheses that (1) ethanol may induce abnormal function very early in cardiac development and (2) that this abnormal function by itself plays a significant role in inducing many of the cardiac defects observed later in development.

### The avian model of fetal acohol syndrome (FAS)

The avian system has been of great value in the analysis of cardiac function during development, because it is accessible for analysis by various tools without compromising the physiological state of the developing embryo (Jenkins et al., 2010a, 2012; Garita et al., 2011; Gu et al., 2011, 2012; Happel et al., 2011; Karunamuni et al., 2014). Placental animal models such as the mouse embryo can be cultured for a limited time *in vitro*, but their function is greatly compromised when the embryo is removed from its *in utero* environment. Echocardiography of embryos of intact pregnant mice or after Caesarian section provides limited information for embryo hearts in the early stages of development and often requires anesthesia of the mother which is known to affect embryonic development (Mazze et al., 1985; Warren et al., 1992). To add to the complexity, anesthesia affects adult mouse cardiovascular function in a strain specific way (Phoon, 2006) and may do the same for embryos and fetuses *in utero* (Huang and Linask, 1998).

Another important advantage of the avian model is that they develop a 4-chambered heart that is similar in many ways in structure and function to the human heart and is much more amenable to experimental intervention without the complicating factor of the maternal environment (Ruijtenbeek et al., 2002). Zebrafish, frogs, and other models that are accessible for analysis at early stages develop fewer cardiac chambers. For the above reasons, we and others use avian embryos to study the etiology of ethanol-induced CHDs.

The etiology of FAS and FASD has been the focus of much study, especially the cellular and molecular mechanisms (e.g., reviewed in Cole et al., 2012; Ungerer et al., 2013; Veazey et al., 2013). Ethanol exposure at gastrulation has been shown to alter the expression of critical proteins (e.g., transcription factors) within the avian cardiac mesoderm at stage 9, before the heart begins to function (Serrano et al., 2010). Thus, there is the potential for ethanol to be affecting early cardiomyocyte differentiation that could potentially affect early cardiac function.

An intriguing hypothesis is that cardiac neural crest cell disturbances that are induced by ethanol (Sulik et al., 1981; Cartwright and Smith, 1995a,b; Rovasio and Battiato, 1995; Chen et al., 2000; Ahlgren et al., 2002; Snider et al., 2007; Flentke et al., 2011; Keyte and Hutson, 2012) can cause early abnormalities in cardiac function (Cartwright and Smith, 1995a; Conway et al., 1997; Creazzo et al., 1998; Waldo et al., 1999; Cavieres and Smith, 2000; Farrell et al., 2001; Li et al., 2003; Hutson and Kirby, 2007; Wentzel and Eriksson, 2009). These disrupted neural crest cells are unable to regulate FGF signaling, leading to abnormal development of the heart tube (Waldo et al., 1999; Hutson and Kirby, 2007) with consequences such as reduced OFT length, looping, and abnormal calcium transients that can affect several functional parameters. While many investigators continue to study molecular pathways that may be leading to ethanol induced defects (Garic-Stankovic et al., 2005; Flentke et al., 2014) and how neural crest cell abnormalities induce CHDs (Hutson and Kirby, 2007; Sato et al., 2011), few if any studies focus on how the functional consequences of ethanol exposure or neural crest disruption promote subsequent steps leading to the fully-fledged post-septation CHDs associated with FAS.

### The study of FAS CHDs

A lack of tools to precisely assess and perturb function early in heart development has hampered the investigation of the influential role of cardiac function in FAS-associated CHDs. The few studies that have measured cardiac function in FAS animal models support the hypothesis that CHDs arising from ethanol exposure could have a significant contribution from abnormal function. Ultrasound has been used to identify abnormal embryonic cardiac function at late stages of mouse development after ethanol exposure at gastrulation (Dlugos and Rabin, 2010; Serrano et al., 2010). Yelin et al. utilized optical imaging and found structural and contraction anomalies in *Xenopus* embryos exposed to alcohol (Yelin et al., 2007). Using cinephotography, other groups observed decreased cardiac output and contractility in chick embryos after ethanol exposure (Ruckman et al., 1988; Bruyere and Stith, 1994). However, although many studies show that altered cardiac function leads to congenital defects (Creazzo et al., 1997; Hogers et al., 1997; Godt et al., 1998; Hogers et al., 1999; Lee et al., 2000; Hove et al., 2003; Reckova et al., 2003; Tai et al., 2005; Lucitti et al., 2006; Atkins and Jain, 2007; Groenendijk et al., 2007, 2008; Keller et al., 2007; Linask and Vanauker, 2007; Vittorini et al., 2007; Yashiro et al., 2007; Chi et al., 2008, 2010; Vermot et al., 2009) and that alcohol creates hemodynamic anomalies (Yelin et al., 2007; Dlugos and Rabin, 2010; Serrano et al., 2010), no studies have addressed whether functional anomalies induced at the level induced by alcohol exposure directly contribute to CHDs. This is due to the fact that there are few imaging techniques that can quantitatively identify rapid functional changes in early embryos and pinpoint the initial responses that can deflect the heart toward a trajectory to CHDs. Our novel set of custom-made optical imaging systems (OCT, OCM, and OM) has allowed us to assay a range of parameters of early cardiac function. In combination with OP to perturb function in a controlled way, we may be able to mimic ethanol-induced functional defects to establish function as a causal factor in the development of ethanol-associated CHDs.

### Application of tools to study the quail model of FAS CHD

For the FAS model that we study, quail embryos are injected with ethanol (40 μl of 50% ethanol in saline) at stage 4–5 (Serrano et al., 2010) during gastrulation. Ethanol dosage was based on previously published protocols (Serrano et al., 2010) as being equivalent to one binge drink episode in humans (4–5 standard drinks on one occasion) and reliably produced FAS-associated CHDs, while allowing survival. Pulsed DOCT traces of the vitelline artery [reflecting cardiac function (Gu et al., 2012)] at HH stage 19 (looping heart) revealed that ethanol-exposed embryos exhibited abnormalities in two parameters (Karunamuni et al., 2014): (1) reduced shoulders and (2) a higher degree of retrograde blood flow in the OFT region compared to controls. Preliminary findings in the dorsal aorta with DOCT also suggested that cardiac output may be reduced in early-stage ethanol-exposed embryos (HH stage 14). Thus, at these very early developmental stages, ethanol exposure has already led to abnormal flow.

In previous studies, altered flow was shown to affect cardiac cushion and valve formation in a zebrafish model (Vermot et al., 2009). At stage 12–19 in the early embryonic quail heart, the superior and inferior atrio-ventricular (AV) cushions have emerged as swellings in the AV canal of the heart, with increased deposition of “cardiac jelly” in these localized regions (Person et al., 2005; Combs and Yutzey, 2009). A subset of endocardial cells lining the cushions will undergo epithelial-to-mesenchymal transition (EMT), release from their neighbors and populate the cushion as proliferating and migrating mesenchymal cells. At this developmental time-point, the AV cushions already serve as physical barriers to minimize regurgitant flow in the early heart tube.

For the study of the quail FAS model, OCT was used to image the embryonic quail heart which at the stages of interest (stage 14–19) is still relatively transparent due to the thin myocardium. 3-D OCT images were obtained of the cardiac cushions, specifically the inferior AV cushion and the contiguous superior atrio-ventricular/conotruncal cushion, and segmented (AMIRA image processing software, FEI Visualization Sciences Group). Cushion volumes were calculated using the Measurement tools in AMIRA. Ethanol-exposed embryos developed smaller cushions compared to saline-treated and untreated controls. Thus at these early stages, reduced cushion sizes and hemodynamic anomalies were already being detected in ethanol-exposed embryos.

At late stages (HH Stage 34; 4-chambered heart), ethanol-exposed quail embryos developed CHDs (valvuloseptal and so called conotruncal defects in proximal and distal portions of the OFT) resembling those found in FAS. The great vessels of the post-septation heart, i.e., the right pulmonary artery (RPA), the left pulmonary artery (LPA), the right brachiocephalic artery (RBA), the left brachiocephalic artery (LBA), and the aortic arch (AA), were imaged using OCT. Use of this technology allowed for rapid phenotyping and efficient cataloging of defects. Prior to imaging, hearts were fixed in formalin and cleared using the Clear-T protocol, involving incubation of the hearts in a series of increasingly concentrated formamide solutions (Kuwajima et al., 2013). This simple clearing process allowed us to image an entire intact heart at stages after ventricular septation (approximately 2–3 mm thick at stage 35) using OCT. These OCT images also allowed for segmentation and quantification of the reduction in valve leaflet volumes as well as revealing the presence of structural defects (see movie Figure S2 in Supplementary Material).

Preliminary findings confirmed that the dose and timing of ethanol administration results in FAS-associated CHDs, thus validating our avian model. Variation in the range of the defects after ethanol exposure will allow us to link severity of early functional and structural parameters with severity of later structural defects in longitudinal studies.

In summary, our avian model for prenatal alcohol exposure developed CHDs by septation stages that were similar to those associated with individuals with FAS. We were able to rapidly identify and assess these CHDs using OCT of cleared hearts at the older stages. Abnormal flow dynamics were detected by OCT at early looping heart stages which may explain the hypoplastic cardiac cushions found in these ethanol-exposed embryos (Karunamuni et al., 2014).

With these biophotonic tools in place, we will now be able to sensitively identify and quantify functional parameters that result from ethanol exposure even from early looping heart stages. In addition, with the advent of OP, we may be able to mimic these abnormal functional parameters in the absence of ethanol exposure to test whether altered cardiovascular function by itself during this vulnerable window of development could play a major role in the progression of ethanol-induced CHDs.

## Future potential

### Functional measurements linked to molecular changes

The biophotonic tools currently available allow us to simultaneously collect data for both function and structure of the embryonic heart. One of the next big challenges is connecting functional parameters with molecular changes in the heart. Analysis of molecular changes either requires the destruction of tissue (e.g., Western blot, PCR) or the processing of tissue (fixation, permeabilizing, clearing) that is not compatible with function. One method of linking these data is to measure the functional parameter in detail and then process the heart immediately to assay for molecular changes by *in situ* hybridization, immunostaining, or other techniques. This information can then be overlaid on a single 3-D data set. This type of dual analysis of embryo hearts has been conducted in zebrafish (e.g., Vermot et al., 2009) and has promise in avian systems (e.g., Groenendijk et al., 2004, 2007; Bressan et al., 2013). For the mouse model, echocardiography of embryos is the technique most often paired with the molecular studies (e.g., Chiplunkar et al., 2013; Rog-Zielinska et al., 2013). The ideal situation would be to have a marker of molecular changes that can be accessed without affecting physiological function. Genetic engineering of Drosophila, zebrafish, mice, and in the future birds specifically for these types of markers may solve this challenge (Wang et al., 2003; Suh et al., 2004; Tallini et al., 2006; Chi et al., 2008).

### OCT for longitudinal studies

With the ability to phenotype the avian heart from early to late stages, a longitudinal study of individual embryos is possible using OCT. This capability would overcome the inevitable variability observed between embryos in stage and degree of abnormality thus reducing the number of embryos required per study. In addition, longitudinal analyses allow more sophisticated statistics (e.g., stratification), thus potentially uncovering links between biomechanical forces and molecular/structural development that are normally hidden in a standard cohort study. As a result, correlation between the level of severity of an early stage functional and structural deficits and the magnitude of later functional and structural defects can be made. For example, we expect to find a graded correlation that ethanol-treated embryos with retrograde flow or OSI in the vitelline vessel close to the normal range will show little change in endocardial cushion volume and valve leaflet size, while elevated retrograde flow significantly above the normal range would result in reduced endocardial cushion volumes and later reduced valve leaflet volumes. This type of analysis would be very difficult with the standard “cohort” designed experiment where embryos at the early developmental stages are sacrificed to make the relevant measurements. Embryos exposed to the same concentration of ethanol could behave very differently as a single cohort, and the live imaging ability of OCT allows us to stratify them based on their early responses yet still allow us to follow their development to observe the later consequences. By these longitudinal analyses “dose responses” to functional parameters could be revealed.

### OCT for mouse embryo heart function

The number of mouse models currently available is enormous and continues to grow each year by the contribution of individual laboratories and by consortia whose goal is to systematically make and characterize mice with mutations spanning the genome (Yu, 2004; Ayadi et al., 2012; Koscielny et al., 2014). While structural and molecular phenotyping of embryos has been carried out on many of these mouse lines, functional parameters have been more difficult to obtain. The study of function of the embryonic murine heart presents a different set of challenges and obstacles when compared to that of embryonic avian heart. Concerted research efforts are needed to find novel approaches to unlock the potential that OCT and other tools can bring to embryonic research utilizing the mouse model.

The *in utero* function of mutant mouse embryo hearts and vessels have been assessed using ultrasound, MRI and CT scanning (Schneider et al., 2003; Kulandavelu et al., 2006; Berrios-Otero et al., 2009, 2012; Liu et al., 2013, 2014b). OCT may complement these techniques. OCT has been used for live imaging of cultured mouse embryos up to E10.5 (Larina et al., 2008, 2009). Mouse embryos can be cultured in a nutrient medium for a couple of days but their cardiac physiology is unlikely to duplicate *in utero* cardiac physiology when separated from the maternal circulation and environment. When the dedidua thins at E12.5, OCT can be used to image the embryo through the uterine wall while the uterus and embryo are still connected to the maternal circulation (Phoon, 2006; Syed et al., 2011). An abdominal incision is made, the uterine horn exteriorized through the incision, and embryos are OCT scanned through the uterine wall. This technique can be used with a few repetitions on the same embryos and allows analysis of limbs, head structures, and brain development. Cardiac structure and function would be difficult to capture at these OCT accessible stages because the growing forelimbs and head of the embryo would impede scanning. With the caveat that this externalizing procedure that includes anesthesia for the pregnant animal may compromise physiological function of the embryo, OCT has the potential to be useful in analysis of flow through accessible embryonic vessels that may reflect cardiac function.

### Conflict of interest statement

The authors declare that the research was conducted in the absence of any commercial or financial relationships that could be construed as a potential conflict of interest.
