# Supplementary material for: Capturing structure and function in an embryonic heart with biophotonic tools
Source: Front Physiol. 2014 Sep 23;5:351. doi: 10.3389/fphys.2014.00351 (PMC4173643; doi:10.3389/fphys.2014.00351)
Supplement: Supplementary file 3 [file Presentation1.PDF]

Supplementary Material

Figure 1S. (Video Clip, opens with Windows Media Player, 5.58 MB) **3-D reconstruction of mouse embryo hearts.** Mouse embryos with ERK genes deleted specifically in neural crest cells exhibited a cardiac phenotype termed persistent truncus arteriosus (PTA) (Newbern et al., 2008). The E16.5 wildtype (left) and knockout (right) mice upper chests were fixed and serially cryosectioned. Digital images of the sections were segmented and reconstructed using AMIRA. In wildtype mice, the aortic (red) and pulmonary (light blue) trunks were separated by this stage. In contrast, the mice with ERK deletions (on the right) had only one trunk (light blue) connected to the ventricles that had failed to separate into the aortic and pulmonary trunks, classic PTA. The right ventricle is light pink and the left ventricle is dark pink. (Newbern et al., 2008).

Figure 2S. (Movie Clip, opens with Windows Media Player, 4.85 MB) **3-D reconstruction of OCT images of a valve leaflet for quantification of volume of a stage 34/35 quail heart**. An atrioventricular valve leaflet (purple) and surrounding tissues (red) were segmented and reconstructed using AMIRA.
